# Colonization of plant roots and enhanced atrazine degradation by a strain of *Arthrobacter ureafaciens*

**DOI:** 10.1007/s00253-017-8405-3

**Published:** 2017-07-12

**Authors:** Dmitry P. Bazhanov, Kai Yang, Hongmei Li, Chengyun Li, Jishun Li, Xiangfeng Chen, Hetong Yang

**Affiliations:** 1 Ecology Institute of Shandong Academy of Sciences, 19 Keyuan Road, Jinan, 250014 Shandong Province People’s Republic of China; 2 Shandong Provincial Analysis and Test Center of Shandong Academy of Sciences, Jinan, Shandong Province People’s Republic of China

**Keywords:** *Arthrobacter*, Root colonization, Atrazine, Degradation products, Soil bioremediation, HPLC-MS/MS

## Abstract

Our previous research found that culturable atrazine degraders associated with maize roots were dominated by genetically similar strains of *Arthrobacter ureafaciens*, suggesting their rhizosphere competence. The present study aimed to assess the root-colonizing capacity of strain *A. ureafaciens* DnL1-1 and to evaluate consequent root-associated degradation of atrazine. A soil-sand assay and pot experiments provided evidence that *A. ureafaciens* DnL1-1 competitively colonized roots of maize, wheat, and alfalfa following seed inoculation. Atrazine was not absolutely required but promoted colonization of plant roots by the bacterium. In association with plants, *A. ureafaciens* DnL1-1 enhanced the degradation of atrazine and strongly reduced accumulation of its dealkylated metabolites. Our results show that after low-level inoculation of seeds, the bacterium *A. ureafaciens* DnL1-1 can establish root populations sufficient for the rapid degradation of atrazine in soil that makes it a promising bioremediation agent which can be easily applied to large areas of polluted soil. Application of the root-colonizing, atrazine-degrading *Arthrobacter* bacteria as seed inoculants may be a reliable remediation strategy for soils contaminated with chlorinated s-triazines and their degradation products.

**Electronic supplementary material:**

The online version of this article (doi:10.1007/s00253-017-8405-3) contains supplementary material, which is available to authorized users.

## Introduction

Strains belonging to the genus *Arthrobacter* are one of the most frequently isolated degraders of atrazine (Arbeli and Fuentes 2010; Krutz et al. 2010; Bazhanov et al. 2016). It is traditionally believed that *Arthrobacter* bacteria, being typical autochthonous soil microbes (Winogradsky 1925) and “K” strategists (Bowen 1980), are associated with bulk rather than rhizosphere soil (Cacciari and Lippi 1987). Nevertheless, some of the early (Cacciari and Lippi 1987) and many more recent investigations (Schweiger and Tebbe 2000; Duineveld et al. 2001; Gomes et al. 2001; Mansfeld-Giese et al. 2002; Polyanskaya et al. 2003; Costa et al. 2006; İnceoğlu et al. 2013) detected abundant populations of *Arthrobacter* bacteria in the rhizosphere of various plants. The observed prevalence of *Arthrobacter* spp. in root-associated microbial communities during the whole growth period or specific stages indicated their rhizosphere competence. However, few researches were done to clarify mechanisms required for rhizosphere growth of *Arthrobacter* strains. So, the hypothesis of efficient nutrient utilization by *Arthrobacter* bacteria during periods of lesser nutrient flux from roots (Herzberg et al. 1978) seemed to be the only trying to explain their rhizosphere-specific abundance.

Genomic analysis of atrazine-degrading *Arthrobacter* strains has shown that the high resistance to environmental stresses such as desiccation, oxidation and osmotic stress, adaptation to nutrient limitations due to metabolic versatility, and long survival time during starvation are the main mechanisms of their survival in soil (Mongodin et al. 2006; Dsouza et al. 2015). Nevertheless, the flagellar operon has been recently found in genome of atrazine-degrading isolate *Arthrobacter* sp. AK-YN10 (Sagarkar et al. 2016). The motility earlier observed in some bacteria of the genus *Arthrobacter* (Antheunisse 1974; Stanlake and Clark 1976) also suggests their active behavior in soil. Krutz et al. (2012) found that the density of atrazine-degrading bacteria in the rhizosphere was nearly twice of that in bulk soil. This suggests that the most successful soil degraders of atrazine are those associated with plant roots and therefore can be expected to actively colonize the rhizoplane and rhizosphere. Our previous research demonstrated that genetically similar atrazine-degrading *Arthrobacter ureafaciens* strains were ubiquitous in exposed to atrazine agricultural soils of Shandong Province and prevailed among atrazine degraders directly isolated from the maize rhizosphere (Bazhanov et al. 2016). The present study aimed to assess the root-colonizing capacity of strain *A. ureafaciens* DnL1-1 applied via inoculation of plant seeds and to evaluate consequent root-associated degradation of atrazine.

## Materials and methods

### Soil

Grassland soil S1 not adapted to atrazine was collected in East Jinan. Location and history of the site and characteristics of S1 soil were previously reported (Bazhanov et al. 2016).

### *s*-triazines

Analytical grade atrazine (#45330 Sigma-Aldrich, Saint-Louis, MO, USA), atrazine-2-hydroxy (HA) (#36631 Sigma-Aldrich, Saint-Louis, MO, USA), atrazine-desethyl (DEA) (#36629 Sigma-Aldrich, Saint-Louis, MO, USA), atrazine-desisopropyl (DIA) (#36628 Sigma-Aldrich, Saint-Louis, MO, USA), and atrazine-desethyl-desisopropyl (DAA) (#36667 Sigma-Aldrich, Saint-Louis, MO, USA) were used as calibration standards and to spike S1 soil samples in extraction recovery experiments. Deuterated atrazine-d5 (^2^H_5_-ATZ) (#34053, Sigma-Aldrich, Saint-Louis, MO, USA), atrazine-2-hydroxy D5 (^2^H_5_-HA) (#DRE-XA10333100ME, LGC Standards, Teddington, UK), atrazine-desisopropyl D5/ethylamino D5/ (^2^H_5_-DIA) (#DRE-C10332100, LGC Standards, Teddington, UK), desethylatrazine-d_7_/*iso*-propyl-d_7_/ (^2^H_7_-DEA) (#D-5639, C/D/N Isotopes Inc., Pointe-Claire, Canada), and ^13^C atrazine-desethyl-desisopropyl (^13^C_3_-DAA) (#CLM-7528-S, Cambridge Isotope Laboratories, Inc., Tewksbury, MA, USA) were used as internal standards at concentrations of 5 ppb (^2^H_5_-ATZ, ^2^H_5_-HA, ^2^H_7_-DEA) and 20 ppb (^2^H_5_-DIA, ^13^C_3_-DAA). The standard stock solutions were prepared in HPLC-grade methanol (Fisher Scientific, Pittsburgh, USA).

Technical atrazine powder (≥97%; Shandong Dehao Chemical Co., Ltd., Weifang, China) was used in the routine growth media for atrazine-degrading bacteria, in soil-sand tube assay, and pot experiments. The high-performance liquid chromatography–tandem mass spectrometry (HPLC-MS/MS) analysis (performed in the manner described below) detected that the technical atrazine contained 0.078 ± 0.002% HA, 0.150 ± 0.003% DEA, 0.120 ± 0.0007% DIA, and 0.010 ± 0.0004% DAA.

### Bacterial strains and growth media

Atrazine-degrading strain *A. ureafaciens* DnL1-1 harboring the genes *trzN* and *atzBC* was earlier isolated from the maize rhizosphere by a direct plating method (Bazhanov et al. 2016). The strain was deposited in the China General Microbiological Culture Collection Center (CGMCC) under the registration no. CGMCC 9667. The type strain *A. ureafaciens* CGMCC 1.1897^T^ was obtained from the CGMCC. Mineral media SM and SMY (Bazhanov et al. 2016) were used to grow atrazine-degrading bacteria. Swimming motility was detected in liquid and semisolid (0.2% agar) medium SA, which contained salts of SM medium and 1 g/L asparagine as sole carbon and nitrogen source.

### Degradation of atrazine and its metabolites by *A. ureafaciens* DnL1-1

The strain *A. ureafaciens* DnL1-1 was grown in liquid medium SM to which stock solutions of analytical grade atrazine, DEA, DIA, and DAA were added separately or in combination instead of the routine atrazine stock suspension. The inoculum was prepared from 3-day cultures on SMY plates at 28 °C. Ten colonies were suspended in 1 mL buffer (SM medium salt solution) and twice washed by centrifugation (3000×*g*, 3 min). A 30 mL fresh medium in 250-mL Erlenmeyer flasks was inoculated 1:1000 with the suspension and incubated at 28 °C without shaking. Growth of the cultures was monitored by plating on R2A agar (#17209 Sigma-Aldrich, Saint-Louis, MO, USA). Samples for HPLC-MS/MS analyses were prepared by mixing equal volumes of culture and methanol followed by removing insoluble material by centrifugation (8000×*g*, 2 min). The mean mass and molar concentrations of the analytes and 95% confidence intervals were calculated based on the analyses of four replicate samples. For graphic representation, the mean colony forming unit (CFU) titers and molar concentrations were transformed to decimal logarithms (lg).

### Motility tests

To detect swimming motility, a 20 mL liquid medium SA in 250-mL Erlenmeyer flasks was inoculated 1:100 with a suspension of *A. ureafaciens* DnL1-1 cells and incubated at 28 °C without shaking. The cultures were examined by light microscopy at 24 h. For semisolid agar assay, a 2 μL suspension of *A. ureafaciens* DnL1-1 was inoculated onto the center of SM and SA semisolid (0.2%) agar plates. The plates were incubated at 28 °C for 2 weeks. Migration of *A. ureafaciens* DnL1-1 along fungal hyphae was detected on soft (0.5% agar) SM medium. A 5-mm plug from the culture of *Rhizoctonia solani* strain 21 (Rovira et al. 1986) on potato dextrose agar (PDA) was transferred to the center of an SM soft agar plate, which was incubated at 28 °C for 2 days until the mycelium reached 20–22-mm diameter. A 10 μL suspension of *A. ureafaciens* DnL1-1 was then inoculated onto the mycelium center (PDA plug). The plates were further incubated for additional 6 days.

### Seed inoculation

A suspension of twice-washed cells of *A. ureafaciens* DnL1-1 was serially diluted from 1:30 to 1:1000 to prepare inocula with various cell densities. For the soil-sand assay, germinated non-sterilized seeds with radicles ≤1 mm were soaked in 30 mL of the diluted bacterial suspensions in Petri dishes for 1 h. Control seeds were soaked in sterile buffer. The seeds were then carefully blotted on sterile filter paper, placed in a Petri dish, and immediately used for the assay.

To determine inoculum level on seeds, 10 wheat seeds or 20 alfalfa seeds were transferred into a 2-mL polypropylene tube with 1 mL buffer, and five maize seeds into a 5-mL polypropylene tube with 2.5 mL buffer. The tubes were secured in a MO BIO Vortex Adapter tube holder assembled on a Vortex-Genie® 2 Vortex (MO BIO Laboratories, Inc., Carlsbad, CA, USA) and vortexed at maximum speed for 10 min. The resulting suspensions and their serial dilutions were plated onto SM agar. Colonies of *A. ureafaciens* DnL1-1 were counted after 4 days at 28 °C. Populations of indigenous seed bacteria were assessed in the non-inoculated control seeds by plating on R2A agar (Sigma-Aldrich, Saint-Louis, MO, USA). Bacterial densities and confidence intervals were calculated according to Koch (1981).

Dry wheat seeds were inoculated in a similar manner, air-dried, and kept at room temperature. Seed population densities of *A. ureafaciens* DnL1-1 were assessed on the day of sowing.

### Soil-sand assay for assessment of root colonization and atrazine degradation

The assay was performed in glass tubes (25 × 200 mm) in the manner described by Scher et al. (1984) with modifications (Supplementary Fig. S1). Each tube contained 33 g of coarse sand moistened with 7 mL of 5 mM KH_2_PO_4_ or atrazine solution in 5 mM KH_2_PO_4_ with a concentration of 0.5, 2.5, or 25.0 μg/mL to obtain herbicide doses of 3.5, 17.5, and 175.0 μg/tube, respectively. The sand was overlaid with 2 g raw S1 soil adjusted to 18% moisture. One inoculated maize or wheat seed or three alfalfa seeds were added per tube. The seeds were covered with another 2 g S1 soil, closed with foil caps, and incubated in a HPG-400 BX growth chamber (Harbin Donglian Electronic Technology Development Co., Ltd., Harbin, China) at 26 °C, 12,000 lx, 12-h day/night cycle. No water was added to the tubes during incubation.

Root colonization was assessed 21–23 days post seeding. Plants were carefully removed from the tubes. All roots were excised under the soil and placed into Petri dishes with 30 mL buffer for 10 min to facilitate detachment of sand grains. Then, the roots were placed on filter paper for 2 s to remove excess moisture and transferred to 2-mL polypropylene tubes with 1 mL buffer. The tubes were secured in a MO BIO Vortex Adapter tube holder assembled on a Vortex-Genie® 2 Vortex (MO BIO Laboratories, Inc., Carlsbad, CA, USA) and vortexed at maximum speed for 10 min to detach root-associated bacteria. The resulting suspensions and their serial dilutions were plated onto SM agar. The washed root sections were blotted and immediately weighed. Mean lg CFU/g fresh root and 95% confidence intervals were calculated based on five replicate tubes for each treatment by using the descriptive statistics tool of MS Excel.

Atrazine degradation in the tubes was assessed 30 days post seeding. The tubes were closed with silicon plugs and stored at −20 °C. The extraction from the whole volume of soil-sand in a tube was performed in the manner described by Krutz et al. (2008). Atrazine was quantified by HPLC-MS/MS, as described below.

### Assessment of root colonization and degradation of atrazine in a soil pot assay

Soil S1 was passed through a 2-mm sieve and weighed (300 g dry weight equivalents) into 500-mL polypropylene pots. Each pot received 70 mL of 5 mM KH_2_PO_4_ or solution of atrazine in 5 mM KH_2_PO_4_ at a concentration of 25.0 μg/mL to provide a dose of 1750 μg/pot that was equivalent to 17.5 kg/ha on the basis of soil weight 3000 t/ha. Seven inoculated or non-inoculated (control) dry wheat seeds were sown to each pot. Additional controls for assessment of atrazine degradation were pots with soil to which no seeds were sown and soil which received inoculated seeds with embryos removed. The pots were placed outdoors and incubated under sunlight with natural photoperiod of Jinan in September to October, day temperatures of 20–26 °C and 15–20 °C at night. During incubation, distilled water was added to the soil along the pot sides using a 10-mL Eppendorf pipette and sterile tips.

Root colonization and degradation of atrazine were assessed 28 days post seeding. Roots were retrieved from the soil cores and cut 15 mm below the seeds. Loosely adhering soil was carefully removed by hand using sterile latex gloves. Then, the roots were processed and root-associated populations were evaluated as described in the “Soil-sand assay for assessment of root colonization and atrazine degradation” section. The soil samples were freeze-dried, homogenized, transferred into polyethylene bags, and kept at −20 °C. Atrazine and its degradation products were extracted from soil and analyzed by HPLC-MS/MS, as described below. Means and 95% confidence intervals for the population densities and amounts of the analytes were calculated based on the results obtained from four replicate pots for each treatment by using the descriptive statistics tool of MS Excel. Molar concentrations of extractable atrazine and its transformation products were calculated to evaluate the total (atrazine plus degradation products) soil contamination.

### BOX-PCR genotyping

Template DNAs and PCR mixtures were prepared as previously described (Bazhanov et al. 2016). The temperature program was as follows: 95 °C for 2 min; then 35 cycles at 94 °C for 30 s, 60 °C for 30 s, and 72 °C for 2 min; and final extension 72 °C for 10 min. The amplification products were analyzed by gel electrophoresis as described before (Bazhanov et al. 2016).

### Extraction of atrazine and its degradation products from soil

Atrazine and its degradation products were extracted from soil by the vortexing method. For extraction, 1 g samples of freeze-dried homogenized soil were weighed into 2-mL tubes and mixed with 1 mL methanol-water solution (4:1 *v*/*v*). The tubes were secured horizontally in a MO BIO Vortex Adapter assembled on Vortex-Genie® 2 Vortex (MO BIO Laboratories, Inc., Carlsbad, CA, USA), vortexed at maximal speed for 1 h, and left in the Vortex Adapter for 16 h. After the incubation, tubes were vortexed for 5 min. The suspension was centrifuged at 8000×*g* for 3 min, and the supernatant was transferred to a 10-mL tube. Then, four additional extractions with 1 mL methanol/water were performed, consisting of 30 min vortexing and centrifugation at 8000×*g* for 3 min. All vortexing steps and the incubation after the first vortexing were performed at 30 °C. The supernatants were combined and analyzed by HPLC-MS/MS as described below.

To evaluate extraction efficiency, dry S1 soil was spiked with 10 and 200 ng/g each of atrazine, HA, DEA, DIA, and DAA. Blank samples of S1 soil were extracted to assess background contamination. The mean percent recoveries of the analytes and 95% confidence intervals at 10 and 200 ng/g were, respectively, 87.7 ± 4.4 and 94.6 ± 5.9 for atrazine, 86.4 ± 2.2 and 86.6 ± 2.8 for HA, 93.7 ± 3.8 and 94.3 ± 1.1 for DEA, 95.6 ± 2.7 and 92.8 ± 1.5 for DIA, and 104.5 ± 4.5 and 99.8 ± 5.7 for DAA.

### HPLC-MS/MS

The culture liquids and soil extracts were analyzed by HPLC-MS/MS on an UltiMate 3000 HPLC system interfaced to a TSQ Vantage triple-quadrupole mass spectrometer (Thermo Fisher Scientific, Waltham, MA, USA). Atrazine and metabolites were separated on a Thermo Scientific^™^ Acclaim^™^ 120 C18 column (250 × 4.6 mm, particle size 5 μm) with a flow rate of 0.6 mL/min. Methanol (A) and 0.1% aqueous formic acid solution (B) were used for HPLC gradient elution. The program of gradient elution was 0 to 6 min, 5% A; 20 to 25 min, 90% A; and 25.1 to 30 min, 5% A. The MS/MS analysis was performed in positive electrospray ionization mode and transitions were measured in multiple reaction monitoring. Parameters of MS/MS and the limits of detection and quantification for the analytes are specified in Table 1.

**Table 1:** MS/MS parameters and the limits of detection (LOD) and quantification (LOQ)

Analytes and internal standards (IS)	Retention time (min)	Precursor ion (*m*/*z*)	Product ions (*m*/*z*)	Collision energies (eV)	Analytical LOD (ng/mL)	Analytical LOQ (ng/mL)	LOD^a^ in soil (ng/g)	LOQ^a^ in soil (ng/g)
Atrazine	24.6	216.0	174.0	18	<0.01	0.05	<0.045	0.225
			104.0	29				
			68.0	34				
^2^H_5_-ATZ (IS)		221.0	179.0	18	n/a	n/a	n/a	n/a
HA	17.4	198.1	156.0	17	<0.01	0.05	<0.045	0.225
			86.0	23				
			69.0	33				
^2^H_5_-HA (IS)		202.7	160.9	18	n/a	n/a	n/a	n/a
			118.9	23				
			69.8	34				
DEA	21.7	188.0	146.0	17	0.02	0.1	0.09	0.45
			104.0	25				
			68.0	30				
^2^H_7_-DEA (IS)		194.7	146.8	19	n/a	n/a	n/a	n/a
			104.7	26				
			79.8	27				
DIA	20.0	174.0	132.0	16	0.2	0.5	0.9	2.25
			104.0	22				
			68.1	29				
^2^H_5_-DIA (IS)		178.6	136.8	19	n/a	n/a	n/a	n/a
			104.8	25				
			68.8	31				
DAA	15.1	146	110.0	16	0.1	0.2	0.45	0.90
			104.0	18				
			68.1	27				
^13^C_3_-DAA (IS)		148.9	113.0	16	n/a	n/a	n/a	n/a

*n/a* not applicable

^a^Indicated LODs and LOQs in soil could be improved by concentration of the extracts

## Results

### Degradation capacity of *A. ureafaciens* DnL1-1

**Fig. 1:**
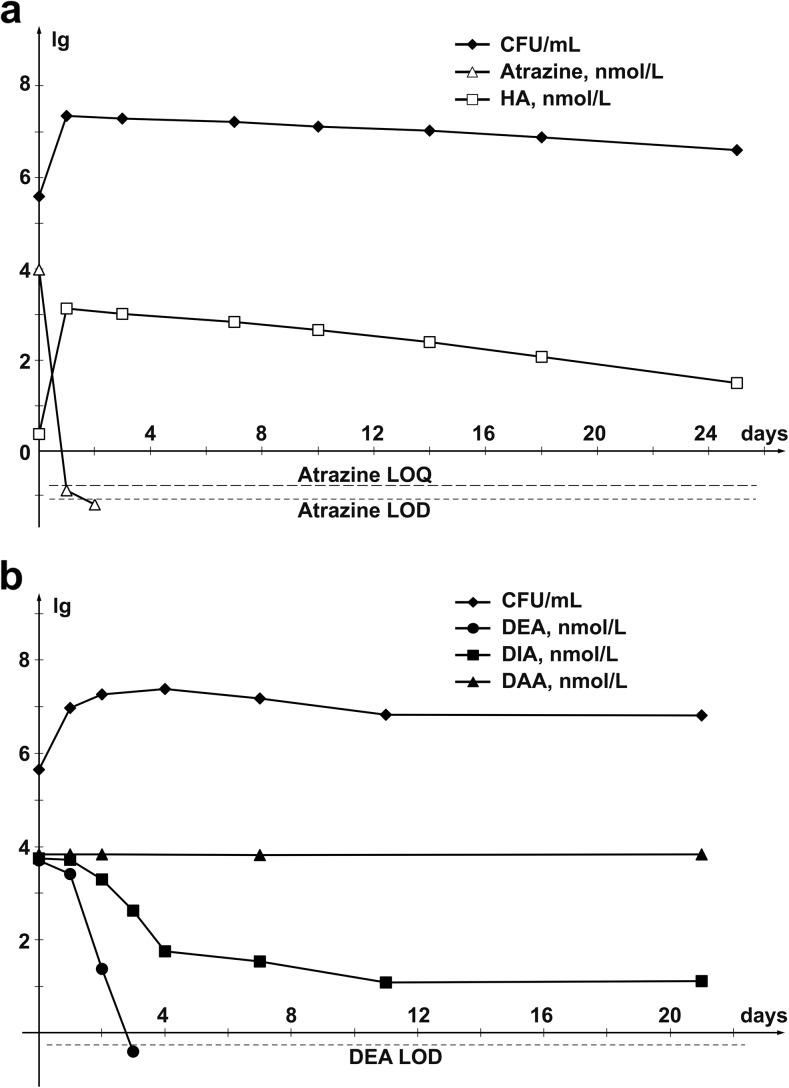
Degradation of **a** atrazine, HA, and **b** dealkylated breakdown products by culture of *A. ureafaciens* DnL1-1

The atrazine-degrading capacity of *A. ureafaciens* DnL1-1 culture was assessed in SM medium containing 2 mg/L atrazine (analytical grade). Trace amounts of the herbicide (≤96.0 ng/L) were detected in the culture liquid 24 h after inoculation (Fig. 1 and Supplementary Table S1). At 48 h, atrazine was not detected in the medium. Nearly 14.0 molar percent of the degraded atrazine were transformed into HA, and the latter was slowly degraded during further incubation of the culture. After 25 days, HA was still detected at a concentration of 5.68 ± 1.22 μg/L, which was equivalent to nearly 0.3% of the initial molar content of atrazine. In the medium supplemented with DEA, DIA, and DAA, each at a concentration of 1.0 mg/L, DEA was not detected after 3-day incubation of *A. ureafaciens* DnL1-1 culture (Fig. 1 and Supplementary Table S2). The content of DIA was reduced nearly 100-fold (to a level of 9.97 ± 1.57 μg/L) during 4 days. After 21 days, DIA was still detected in the medium at a concentration of 2.26 ± 0.20 μg/L (nearly 0.23% of the initial DIA content). No degradation of DAA by *A. ureafaciens* DnL1-1 was observed during 3-week incubation (Fig. 1 and Supplementary Table S2).

### Motility and migration of *A. ureafaciens* DnL1-1

**Fig. 2:**
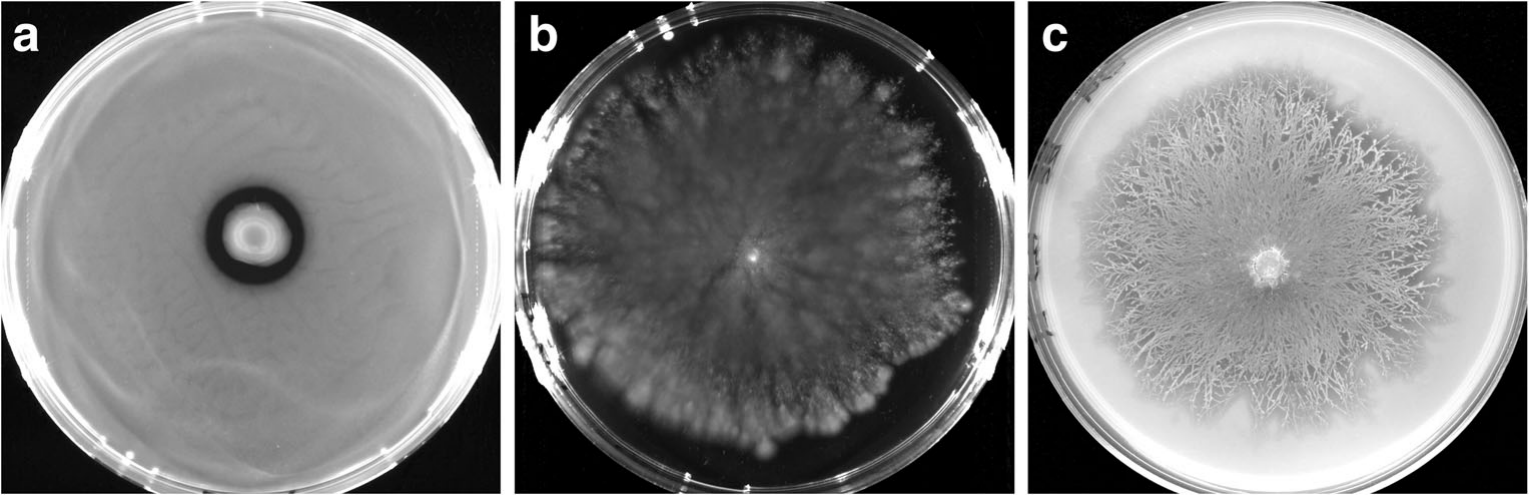
Colony patterns of *A. ureafaciens* DnL1-1 in semisolid media **a** SM, **b** SA, and **c** migration of the bacterium along hyphae of *R. solani* strain 21

All tested 24-h cultures of *A. ureafaciens* DnL1-1 in liquid SA medium were rods, some of which exhibited rapid directional movement. The colony of *A. ureafaciens* DnL1-1 expanded in semisolid (0.2% agar) SA medium, reaching the plate edge within 14 days (Fig. 2). No active migration of *A. ureafaciens* DnL1-1 was detected in semisolid SM agar. On soft SM agar, strain DnL1-1 readily colonized hyphae of *R. solani* strain 21 following inoculation of the mycelium center (Fig. 2).

### Root colonization by *A. ureafaciens* DnL1-1 in a soil-sand assay

Colonization of maize, wheat, and alfalfa roots by *A. ureafaciens* DnL1-1 was evaluated at high and low inoculum levels on seeds (Table 2). Indigenous bacterial populations of germinating seeds reached 8.2 ± 0.1 lg CFU per maize seed, 7.1 ± 0.1 lg CFU per seed of wheat, and 6.3 ± 0.1 lg CFU per alfalfa seed. Seed populations of *A. ureafaciens* DnL1-1 at high inoculum levels were nearly equal to or several times lower than the indigenous populations of maize and wheat seeds and did not exceed 1% of the total bacteria on alfalfa seeds (Table 2). At low inoculum levels, the density of *A. ureafaciens* DnL1-1 seed populations was reduced 100–1000-fold compared to the high inoculum levels.

**Table 2:** Seed and root populations of *A. ureafaciens* DnL1-1 in soil-sand tube and soil pot assays

Assay	Plant	Atrazine dose	Seed population (lg CFU/seed)	Root population (lg CFU/g fresh root)
Soil-sand tube	Maize	None	None	<2.04
			4.23 ± 0.03	<1.66–3.68
			7.87 ± 0.02	3.32 ± 0.86
		175.0 μg/tube	None	<2.0
			4.23 ± 0.03	5.25 ± 0.73
			7.87 ± 0.02	5.88 ± 0.51
	Wheat	None	None	<1.70
			3.45 ± 0.04	<1.56–2.51
			6.67 ± 0.02	4.43 ± 0.50
		3.5 μg/tube	None	<2.46
			3.45 ± 0.04	3.16 ± 0.35
			6.67 ± 0.02	4.95 ± 0.97
		175.0 μg/tube	None	<2.07
			3.45 ± 0.04	6.17 ± 0.23
			6.67 ± 0.02	6.26 ± 0.41
	Alfalfa	None	None	<2.64
			2.08 ± 0.04	<2.81
			4.32 ± 0.02	<2.75–2.84
		17.5 μg/tube	None	<2.72
			2.08 ± 0.04	4.59 ± 0.56
			4.32 ± 0.02	5.34 ± 0.19
Soil pot	Wheat	None	None	<1.70
			4.81 ± 0.02	3.47 ± 0.50
		1750 μg/pot	None	<2.18
			4.81 ± 0.02	6.04 ± 0.24

**Fig. 3:**
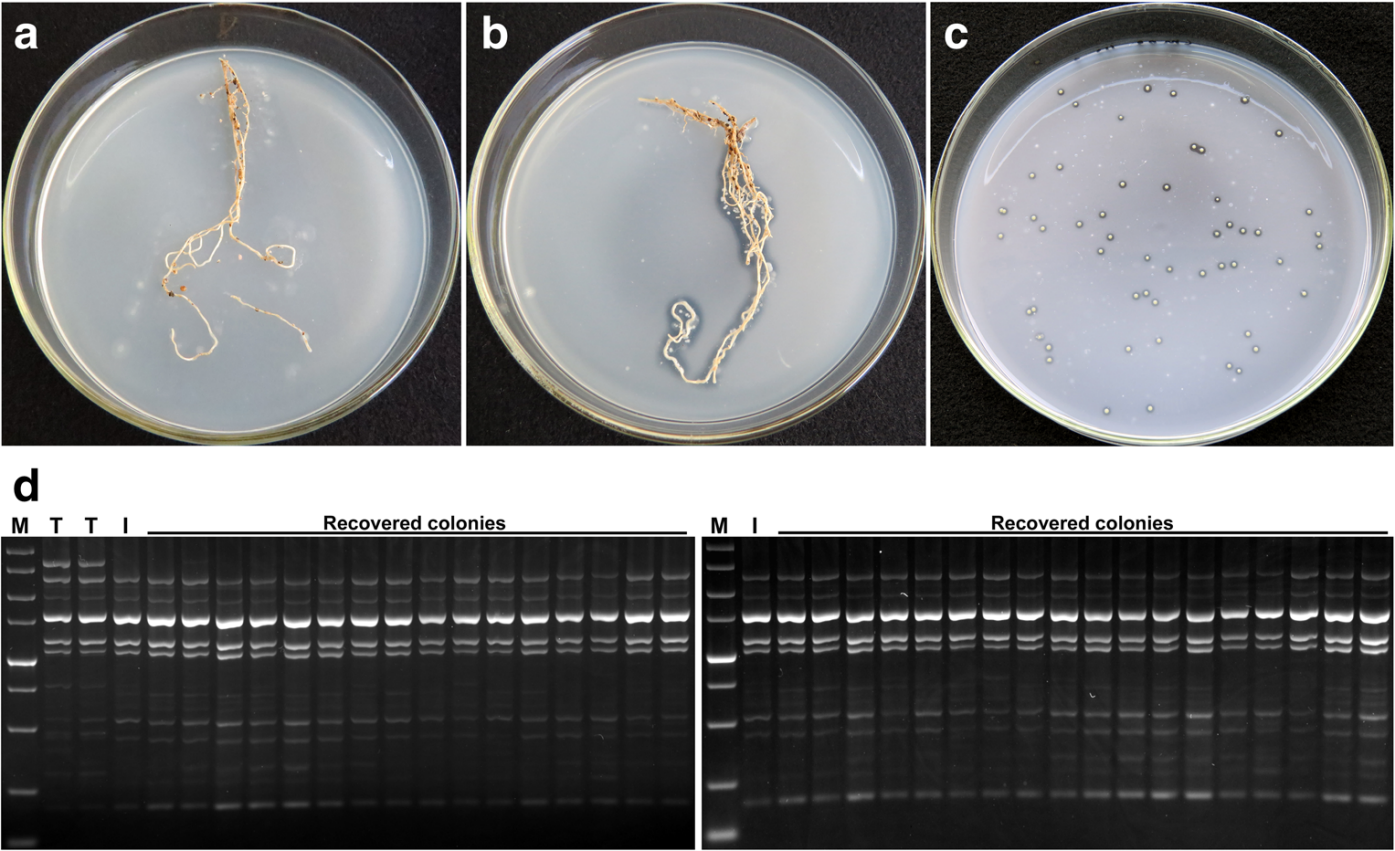
Recovery of *A. ureafaciens* DnL1-1 from wheat roots. **a** Roots of a control uninoculated plant. **b** A zone of atrazine degradation (clearing halo) in SM agar near roots colonized by *A. ureafaciens* DnL1-1 and **c** recovery of atrazine-degrading colonies. **d** BOX-PCR banding patterns of the recovered colonies. *Lanes T* patterns of the type strain *A. ureafaciens* CGMCC 1.1897^T^. *Lanes I* patterns of the inoculant strain *A. ureafaciens* DnL1-1. *Lanes M* contain a DL 5000 DNA marker (TaKaRa Bio Inc.)

As a preliminary check for the presence of root-associated atrazine degraders, the root systems of 3-week-old wheat plants were placed on SM agar and incubated at 28 °C for 2 weeks. Clearing halos near roots of the inoculated plants became visible along the entire root length on the third day (Fig. 3). No atrazine dissolution was detected near or under the roots of control non-inoculated plants during the whole observation period.

In the tubes containing 175 μg atrazine, recovered populations of *A. ureafaciens* DnL1-1 were above 5 lg CFU/g fresh root of maize and above 6 lg CFU/g fresh root of wheat regardless of the inoculum level (Table 2). Lower population densities of *A. ureafaciens* DnL1-1 were observed on wheat roots at an atrazine dose of 3.5 μg/tube. However, the differences detected were statistically significant (*P* < 0.05) only at the low seed inoculum level. The density of *A. ureafaciens* DnL1-1 population associated with alfalfa roots was over 5 lg CFU/g fresh root at the high inoculum level and an atrazine dose of 17.5 μg/tube (Table 2). Inoculation of alfalfa seeds with more than 100-fold reduced density resulted in somewhat lower root populations of the atrazine degrader.

At zero dose of atrazine, stable colonization of maize and wheat roots by *A. ureafaciens* DnL1-1 was observed only after the high-density inoculation of seeds. However, population densities of *A. ureafaciens* DnL1-1 on the roots were nearly 100 times lower than those detected at the high atrazine dose. At the low inoculum levels and zero atrazine dose, root-associated populations of *A. ureafaciens* DnL1-1 were close to the detection limit or even not detected in some tubes.

No atrazine degraders were detected on the roots of non-inoculated control plants at any atrazine dose. The authenticity of atrazine-degrading colonies recovered from roots of the inoculated plants was examined by BOX-PCR. The banding patterns of all the isolates tested (a total of 56 independent isolates) were identical to those of *A. ureafaciens* DnL1-1 (Fig. 3).

### Degradation of atrazine by *A. ureafaciens* DnL1-1 in a soil-sand assay

Atrazine damage became clearly visible in non-inoculated alfalfa and (at the 175 μg/tube atrazine dose) wheat seedlings 20–24 days post seeding. Development of the injury symptoms resulted in plant death by the end of the incubation (Supplementary Fig. S1). The low (3.5 μg/tube) dose did not damage wheat plants incubated under artificial light in a growth chamber. No atrazine injury occurred in wheat plants inoculated by *A. ureafaciens* DnL1-1. The high inoculant dose eliminated atrazine injury symptoms in alfalfa, while slight growth inhibition still occurred after the low-level inoculation (Supplementary Fig. S1).

**Fig. 4:**
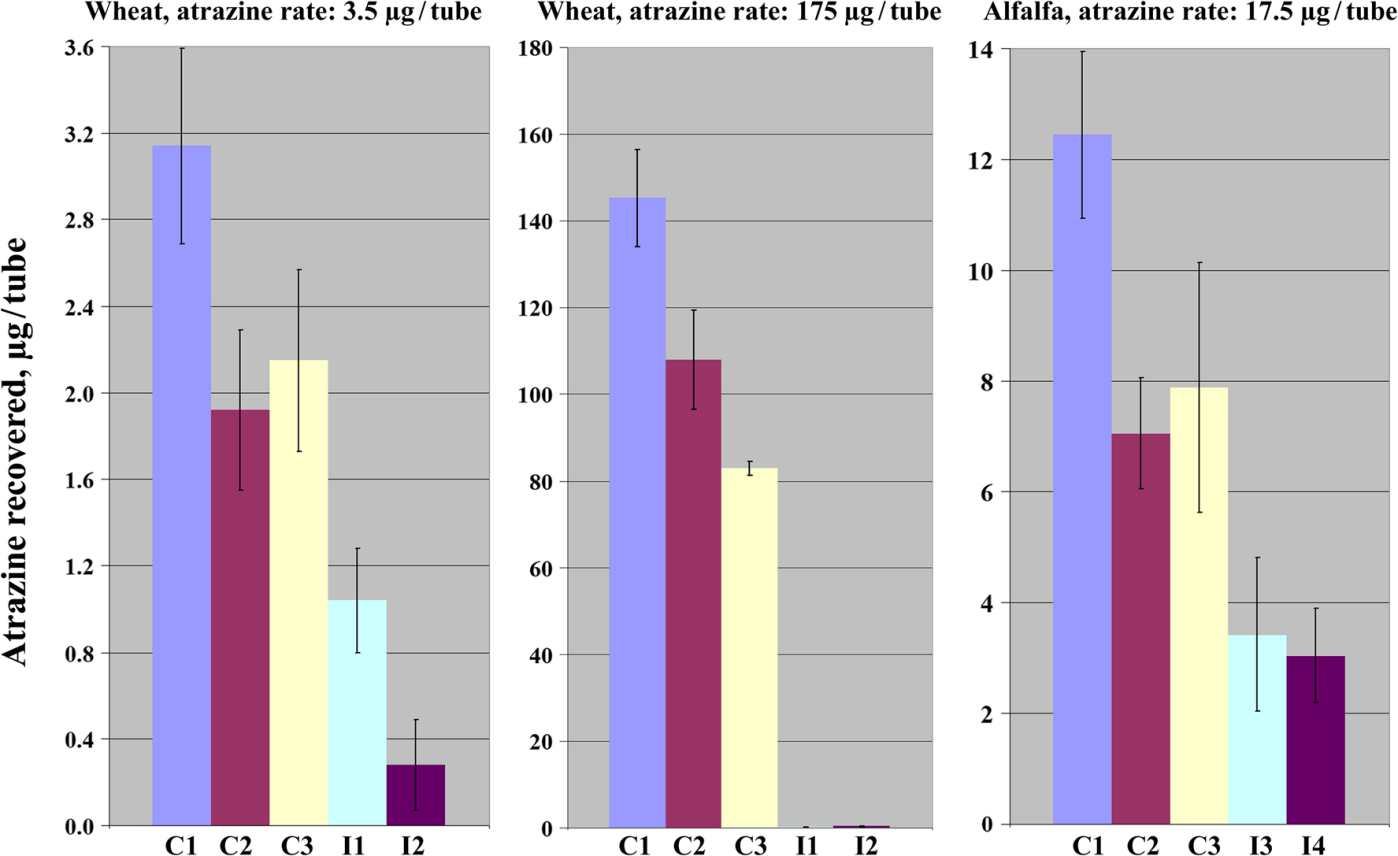
Root-associated degradation of atrazine by *A. ureafaciens* DnL1-1 in a soil-sand tube assay. Inoculum levels 3.45 ± 0.04 lg CFU/seed (I1), 6.67 ± 0.02 lg CFU/seed (I2), 2.08 ± 0.04 lg CFU/seed (I3), 4.32 ± 0.02 lg CFU/seed (I4). Controls: not incubated tubes (C1), not inoculated not planted tubes (C2), not inoculated planted tubes (C3)

Analysis performed after 30 days of incubation demonstrated a profound effect of seed inoculation with *A. ureafaciens* DnL1-1 on atrazine degradation. At the high dose of the herbicide (175.0 μg/tube), its degradation by *A. ureafaciens* DnL1-1 in association with wheat roots was nearly complete (up to 99.8%), regardless of the density of seed inoculation (Fig. 4 and Supplementary Table S3). Approximately 91.8% of atrazine were degraded by the wheat-bacterium association in the tubes with low (3.5 μg/tube) atrazine dose and high density of seed inoculation. As a result, the contents of residual atrazine in the tubes with inoculated wheat plants that had received the high and low herbicide doses were similar (less than 0.5 μg/tube; Supplementary Table S3). Significantly higher residual contents of atrazine (1.04 ± 0.25 μg/tube) were measured at the low atrazine dose and low level of seed inoculum (Fig. 4 and Supplementary Table S3).

Up to 75.6% of the applied atrazine were degraded by *A. ureafaciens* DnL1-1 in association with alfalfa (Fig. 4 and Supplementary Table S3). No significant effect of the inoculation density on atrazine degradation was detected.

Atrazine degradation in the incubated controls ranged from 25.7 to 43.3% and was always significantly lower than in the inoculated tubes. At the high atrazine dose (175.0 μg/tube), its residues in the tubes with non-inoculated wheat plants were lower than in the control non-planted tubes. No plant effect on the level of residual atrazine was detected at a dose of 3.5 μg/tube for wheat and 17.5 μg/tube for alfalfa.

### Colonization of wheat roots by *A. ureafaciens* DnL1-1 and degradation of atrazine in a soil pot assay

The indigenous population on the dry wheat seeds averaged 2.28 ± 0.05 lg CFU/seed that was less than 1% of the inoculant population (Table 2). Atrazine damage resulted in the death of all non-bacterized plants in control pots after 3-week incubation. Seed inoculation with *A. ureafaciens* DnL1-1 reduced herbicide damage and the bacterized wheat plants remained alive till the end of the experiment despite significant injury symptoms occurred. Twenty-eight days post seeding, root populations of *A. ureafaciens* DnL1-1 reached ca. 6 lg CFU/g fresh root in the atrazine-supplemented soil and 10- to 100-fold lower densities at zero atrazine dose (Table 2). All recovered atrazine degraders had similar colony morphology and produced BOX-PCR banding patterns similar to those of *A. ureafaciens* DnL1-1 (a total of 32 isolates were genotyped). No atrazine-degrading bacteria were detected on roots of control uninoculated plants both in the atrazine-supplemented and non-supplemented soils.

HPLC-MS/MS analysis revealed minor contamination of blank S1 soil with atrazine, HA, DEA, DIA, and DAA (Table 3). The atrazine content in atrazine-supplemented S1 soil averaged 5911.4 ± 518.0 μg/kg, indicating that the extraction recovery was 101.3 ± 8.9%. The concentrations of HA, DIA, and DEA in the atrazine-supplemented S1 soil exceeded background levels by nearly 70, 80, and 130 times, respectively, and the content of DAA was about twice the background level. While the amount of DAA measured was close to the expected level (a sum of DAA in blank soil and DAA in atrazine dose applied), the concentrations of HA, DIA, and DEA were much higher than expected (Table 3).

**Table 3:** Quantification of atrazine and its degradation products in soil

Analyte	Dose applied (ng/g dry soil)	Analyte concentrations in soils (ng/g dry soil)						
		Blank soil	Blank soil + atrazine (expected)	Blank soil + atrazine (measured)	Uninoculated unplanted control	Uninoculated planted (wheat) control	Inoculated unplanted control	*A. ureafaciens* DnL1-1 in association with wheat
Atrazine	5833.3	0.99 ± 0.13	5834	5911.4 ± 518.0	3192.7 ± 387.5	3404.7 ± 272.7	2404.1 ± 480.5	125.1 ± 8.4
HA	4.55 ± 0.12	0.98 ± 0.05	5.55	67.7 ± 2.8	198.3 ± 15.0	226.1 ± 44.4	166.6 ± 19.6	151.7 ± 8.6
DEA	8.75 ± 0.18	0.47 ± 0.04	9.25	63.2 ± 1.5	1029.2 ± 114.6	975.4 ± 60.5	440.1 ± 36.9	17.2 ± 2.3
DIA	7.00 ± 0.04	0.25 ± 0.01	7.25	20.6 ± 0.9	219.0 ± 18.5	234.3 ± 11.6	122.3 ± 12.3	17.6 ± 1.6
DAA	0.58 ± 0.02	0.42 ± 0.08	1.00	0.90 ± 0.03	186.8 ± 15.6	178.1 ± 6.9	60.9 ± 9.1	41.3 ± 1.8

After 28-day incubation, the degradation of atrazine in non-planted and planted control soils amounted to 42–46% (Table 3). The analysis of atrazine metabolites in the control soils revealed accumulation of DEA as a major breakdown product and HA, DIA, and DAA as minor ones. Seed inoculation with *A. ureafaciens* DnL1-1 and the resultant colonization of roots led to the degradation of nearly 97.9% of the herbicide applied, prevented the accumulation of DIA, and reduced DEA concentration by 3.7 times compared to its level in the non-incubated atrazine-supplemented soil. The accumulation of DAA in soil that harbored bacterized wheat plants was reduced by more than four times, and the content of HA was reduced by nearly 25–33% compared to the incubated non-planted and planted controls.

Only about 59% of the atrazine were degraded in soil that received the inoculum of *A. ureafaciens* DnL1-1 on the non-viable wheat seeds (Table 3), while the contents of DEA, DIA, and DAA were higher by 25, 7, and 1.5 times, respectively, compared to those in soil with inoculated wheat plants. The content of HA in the inoculated planted and plantless soils was similar.

**Fig. 5:**
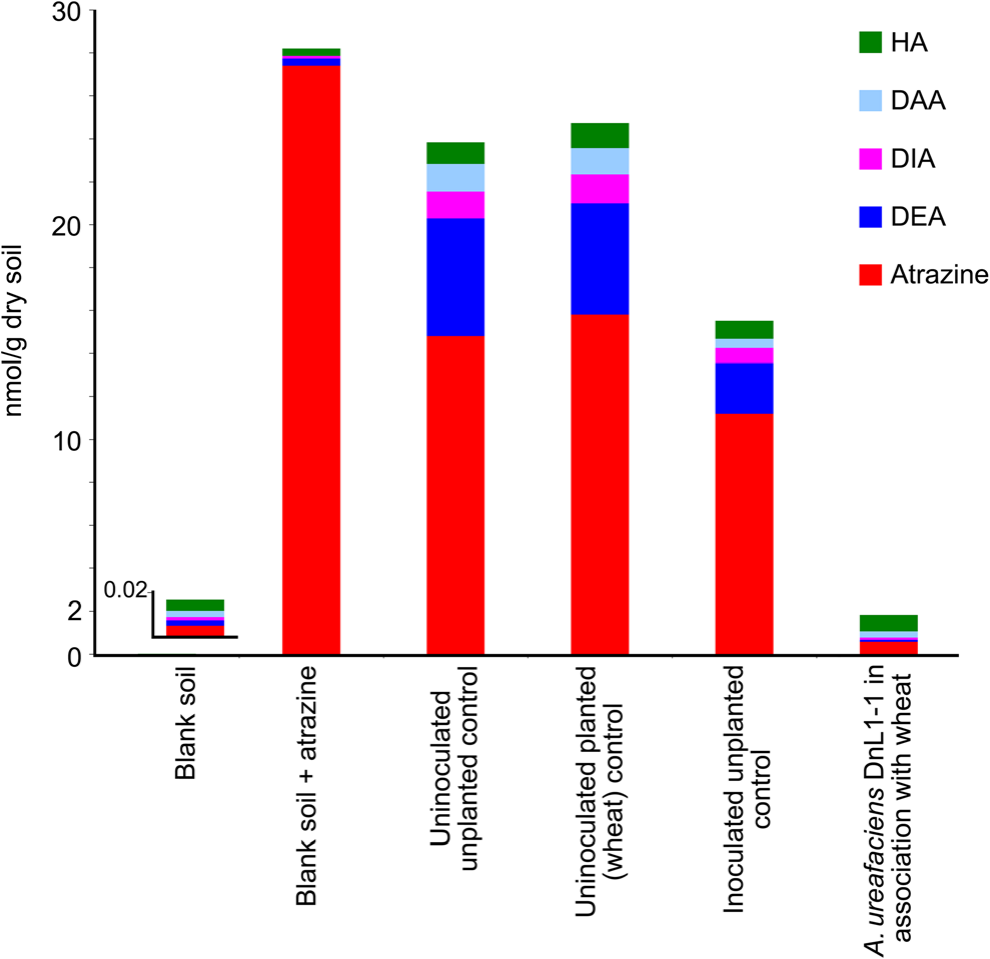
Molar balance of atrazine and its degradation products in soil

The total molar amount of extractable atrazine and its degradation products in the uninoculated non-planted and planted control soils reached 23.84 ± 2.71 and 24.72 ± 1.96 μmol/kg, respectively, or about 85% of their initial total amount (28.21 ± 2.43 μmol/kg) in the atrazine-supplemented soil (Fig. 5 and Supplementary Table S4). The total contamination of soil that harbored *A. ureafaciens* DnL1-1 in association with wheat plants was 1.83 ± 0.12 μmol/kg or nearly 6.5% of the initial level. The total yield of the analytes from the soil to which *A. ureafaciens* DnL1-1 was delivered on the non-viable seeds amounted to 15.52 ± 2.63 μmol/kg (nearly 55% of the initial level).

## Discussion

It is known that the ability to competitively grow on roots in the presence of soil microbes is a key criterion to recognize the root-colonizing bacteria (Kloepper and Beauchamp 1992). Therefore, the availability of a method for differentiating the introduced bacterium from indigenous microorganisms is essential for measuring its root-colonizing capacity. Unlike other known media for the selective isolation of atrazine degraders, SM agar enables their rapid direct recovery from field soil (Bazhanov et al. 2016), thus making possible the quantification of *A. ureafaciens* DnL1-1 in microbial communities harboring no other atrazine-degrading bacteria capable of growing on this medium.

This study provides evidence that *A. ureafaciens* strain DnL1-1 competitively colonizes plant roots following inoculation of seeds. Clearing halos produced in SM agar near the roots indicated that *A. ureafaciens* DnL1-1 migrated to the entire root system. In the soil-sand assay, the bacterium was recovered from roots of maize, wheat, and alfalfa, showing no strong “host” specificity. Scher et al. (1984) demonstrated that the assay enabled consistent assessment of the root-colonizing capacity due to competition of the test bacterium with indigenous soil microorganisms. Because no water is added to tubes during a soil-sand assay, the passive transfer of inoculant cells with the water flow is minimized, and active motion is required for migration of the tested bacteria on roots.

At the high inoculum levels and zero dose of atrazine, the densities of *A. ureafaciens* DnL1-1 root populations were within the range reported by Scher et al. (1984) for strains of fluorescent pseudomonads tested in a soil-sand assay under similar inoculation parameters and incubation conditions. This suggests that the root-colonizing capacity of *A. ureafaciens* DnL1-1 is comparable to those of fluorescent pseudomonads considered to be excellent root colonizers (Lugtenberg et al. 2001). Root populations of *A. ureafaciens* DnL1-1 at zero atrazine dose and low-level seed inoculation were close to the detection limit, indicating that the initial relative abundance of the introduced bacterium was critical for its rhizosphere growth. Such a result was not unexpected, considering large populations of indigenous seed and soil bacteria and assuming that a large portion of these were rhizosphere competent. A direct positive relationship between inoculum size on seeds and root population density was demonstrated for root-colonizing strains of fluorescent pseudomonads (Bull et al. 1991; Raaijmakers and Weller 2001); therefore, higher doses of inoculant on seeds were required to maintain root populations.

An atrazine dose as low as 3.5 μg/tube facilitated the colonization of wheat roots by *A. ureafaciens* DnL1-1 following low-level seed inoculation, although the density of root populations of the colonizer still depended on the inoculum size. At the high atrazine dose, *A. ureafaciens* DnL1-1 reached the highest root populations similarly at both high and low levels of seed inoculation. The ability to establish root populations of a high density regardless of the density of seed population was so far found only in superior root colonizers (Raaijmakers and Weller 2001). The root-colonizing capacity of *A. ureafaciens* DnL1-1 observed in the pot experiment was similar to that determined in the soil-sand assay. Thus, both assays provided evidence that atrazine enhanced the competitiveness of *A. ureafaciens* DnL1-1 in the rhizosphere.

Numerous investigations have demonstrated that motility and chemotaxis are essential for bacterial colonization of plant roots (Lugtenberg et al. 2001; Compant et al. 2010). Our results clearly indicate that *A. ureafaciens* strain DnL1-1 exhibits swimming motility and chemotaxis toward asparagine. The observed migration along fungal hyphae is an example of *A. ureafaciens* DnL1-1 active behavior. The active migration may be also important for colonization of plant roots by *A. ureafaciens* DnL1-1. Studying synthesis of flagella in *Arthrobacter atrocyaneus*, *Arthrobacter citreus*, and *Arthrobacter symplex*, Stanlake and Clark (1976) found that motility was repressed in coccoidal cells for all the species and began only after induction to the rod morphology. Therefore, at least two mechanisms can be proposed by which atrazine favors the colonization of roots by *A. ureafaciens* DnL1-1. It is clear that the nutritional gains from catabolism of atrazine could provide a selective advantage to *A. ureafaciens* DnL1-1 in the rhizosphere, directly promoting growth of the population. In its turn, the activation of *A. ureafaciens* DnL1-1 cell growth might induce production of the motile rods, thus facilitating migration of the bacterium on roots.

Describing Bowen’s (1980) "K" and “r” strategies of bacterial survival in soil, Lynch (1984) suggested that “… the completely successful soil organism will certainly exhibit characteristics of both types of ecological adaptation.” The recently observed influence of plant species and/or growth stage on the rhizosphere abundance of *Arthrobacter* bacteria (Costa et al. 2006; İnceoğlu et al. 2013; Schweiger and Tebbe 2000) indicates their fast response to changing environmental conditions and suggests that arthrobacter’s secrets of soil survival are not limited to the K strategy and stress tolerance mechanisms. Our study provides support to the idea that active behavior and zymogenous response can contribute to the ecological success of *Arthrobacter*. In the absence of atrazine, strain *A. ureafaciens* DnL1-1 competitively colonized plant roots, demonstrating spatial distribution although still remaining a minor component of the rhizosphere microbial community. In atrazine-supplemented soil, *A. ureafaciens* DnL1-1 established large root populations regardless of the inoculum size, exhibiting strong traits of the r strategist.

The analysis of atrazine residues after growth of bacterized root systems provided evidence that inoculation of plant seeds with *A. ureafaciens* DnL1-1 had a substantial effect on the fate of the herbicide by enhancing its degradation. In a soil-sand assay with wheat plants, the amount of atrazine was reduced to less than 0.5 μg/tube in all treatments where seed inoculation resulted in the establishment of large (ca. 5 to 6 lg CFU/g root) populations of *A. ureafaciens* DnL1-1. The content of residual atrazine was significantly higher (1.04 ± 0.25 μg/tube) where the degrader’s populations on wheat roots were lower than 4 lg CFU/g, indicating that *A. ureafaciens* DnL1-1 played a key role in atrazine degradation. Association of *A. ureafaciens* DnL1-1 with alfalfa plants also exhibited enhanced degradation of atrazine. However, despite successful colonization of alfalfa roots by strain DnL1-1, the level of atrazine residues was significantly higher than that detected in tubes which harbored wheat-*A. ureafaciens* DnL1-1 associations, indicating that the "host" plant affected the extent of atrazine degradation.

Root populations established by *A. ureafaciens* DnL1-1 on wheat in the soil pot assay were sufficient to degrade almost all of the atrazine applied to soil. In contrast, the level of atrazine residues in soil received *A. ureafaciens* DnL1-1 on non-viable wheat seeds was significantly higher and comparable to that in the control non-inoculated soil. This indicated that the high atrazine-degrading activity of *A. ureafaciens* DnL1-1 was associated with plant roots.

In both the soil-sand and soil assays, a significant portion of atrazine (42–46%) was degraded in the control planted and plantless treatments not inoculated with *A. ureafaciens* DnL1-1. The S1 soil used in the soil-sand assay and pot experiments had not been exposed to atrazine, and no bacteria capable of rapid atrazine degradation could be isolated from it either directly or after enrichment (Bazhanov et al. 2016). The observed accumulation of DEA as a major degradation product and DIA and DAA as minor ones in the control treatments clearly demonstrated degradation of atrazine through the dealkylation pathway intrinsic to *s*-triazine non-adapted soils (Krutz et al. 2010). As a result, the total contamination by atrazine and its dealkylated products was only slightly reduced.

Seed inoculation and subsequent colonization of roots by *A. ureafaciens* DnL1-1 enhanced the degradation of atrazine and changed the formation of atrazine metabolites in soil. HA was the main degradation product detected in the soils harbored wheat-*A. ureafaciens* DnL1-1 association; however, its accumulation was reduced compared to the control non-inoculated treatments. The contents of DEA and DIA were below their initial levels measured in the atrazine-supplemented S1 soil and the accumulation of DAA was reduced. Because no DAA-degrading activity was detected in pure culture of *A. ureafaciens* DnL1-1, the latter suggested that rapid degradation of atrazine by the introduced bacterium prevented its dealkylation by indigenous microorganisms.

Thus, the results of our study provide evidence that the root-colonizing capacity of atrazine-degrading strain *A. ureafaciens* DnL1-1 facilitates fast adaptation of planted soil to the herbicide. It is known that the efficacy of weed control by *s*-triazine herbicides can be significantly reduced in the adapted soils (Krutz et al. 2010). Almost complete elimination of atrazine injury symptoms in wheat and alfalfa observed in the soil-sand assay under artificial light and prevention of wheat seedling death in the pot experiment, where sunlight enhanced atrazine toxicity, indicated that inoculation of seeds with *A. ureafaciens* DnL1-1 significantly reduced plant exposure to the herbicide. The observed wide "host" range of the strain suggested that colonization of weed roots by atrazine-degrading *A. ureafaciens* might contribute to their resistance to the herbicide in field. On the other hand, seed inoculation followed by colonization of plant roots is an effective method of delivering *A. ureafaciens* DnL1-1 to contaminated soil. It is known that application of *s*-triazine-degrading bacteria for soil bioremediation requires high doses (from at least 5 to 7 lg CFU/g dry soil) of inoculants (Topp 2001; Morgante et al. 2010; Wang et al. 2013; Sagarkar et al. 2016), repeated treatments (Newcombe and Crowley 1999; Morgante et al. 2010), and addition of carbon and energy sources to the soil for stimulation of the atrazine-degrading activity (Mandelbaum and Wackett 1996). Owing to its root-colonizing ability, *A. ureafaciens* DnL1-1 can be introduced to atrazine-contaminated soil by a single low-dose treatment of seeds and can establish root populations sufficient to effectively degrade atrazine and reduce concentrations of its dealkylated metabolites. This makes strain *A. ureafaciens* DnL1-1 a promising bioremediation agent which can be easily applied to large areas of polluted soil.

The study reported here is the first to demonstrate the root-colonizing ability in an atrazine-degrading soil bacterium. Selective advantages from utilization of atrazine enhanced the root-colonizing capacity of the investigated strain *A. ureafaciens* DnL1-1, but were not absolutely required for the competitive colonization of plant roots. Our findings provide evidence that *A. ureafaciens* DnL1-1 can rapidly respond to favorable environmental conditions and establish large root populations after the low-level inoculation, indicating that the r strategy of adaptation to soil environment and active behavior are intrinsic to at least some representatives of the genus *Arthrobacter* and can contribute to their environmental success. The ability to colonize plant roots and high atrazine-degrading capacity in soil makes *A. ureafaciens* DnL1-1 a promising model for studying agronomic and ecological implications of the root-associated atrazine degradation. The release of *A. ureafaciens* DnL1-1 through inoculation of plant seeds is a convenient method to deliver the atrazine degrader to large areas of contaminated soil.

## Electronic supplementary material

ESM 1 (PDF 316 kb)
