# Supplementary material for: Colonization of plant roots and enhanced atrazine degradation by a strain of Arthrobacter ureafaciens
Source: Appl Microbiol Biotechnol. 2017 Jul 12;101(17):6809–20. doi: 10.1007/s00253-017-8405-3 (PMC5554279; doi:10.1007/s00253-017-8405-3)
Supplement: Supplementary file 1 — (PDF 316 kb) [file 253_2017_8405_MOESM1_ESM.pdf]

Applied Microbiology and Biotechnology

Colonization of plant roots and enhanced atrazine degradation by a strain of *Arthrobacter ureafaciens*

Dmitry P Bazhanov[1], Kai Yang[1], Hongmei Li[1], Chengyun Li[1], Jishun Li[1], Xiangfeng Chen[2], Hetong Yang[1]

[1] Ecology Institute of Shandong Academy of Sciences, Jinan, Shandong Province, P. R. China
[2] Shandong Provincial Analysis and Test Center of Shandong Academy of Sciences, Jinan, Shandong Province, P. R. China

Corresponding author: Dmitry P Bazhanov, Ecology Institute of Shandong Academy of Sciences, 19 Keyuan Road, Jinan, 250014, Shandong Province, P. R. China.
E-mail: bazhdp@outlook.com

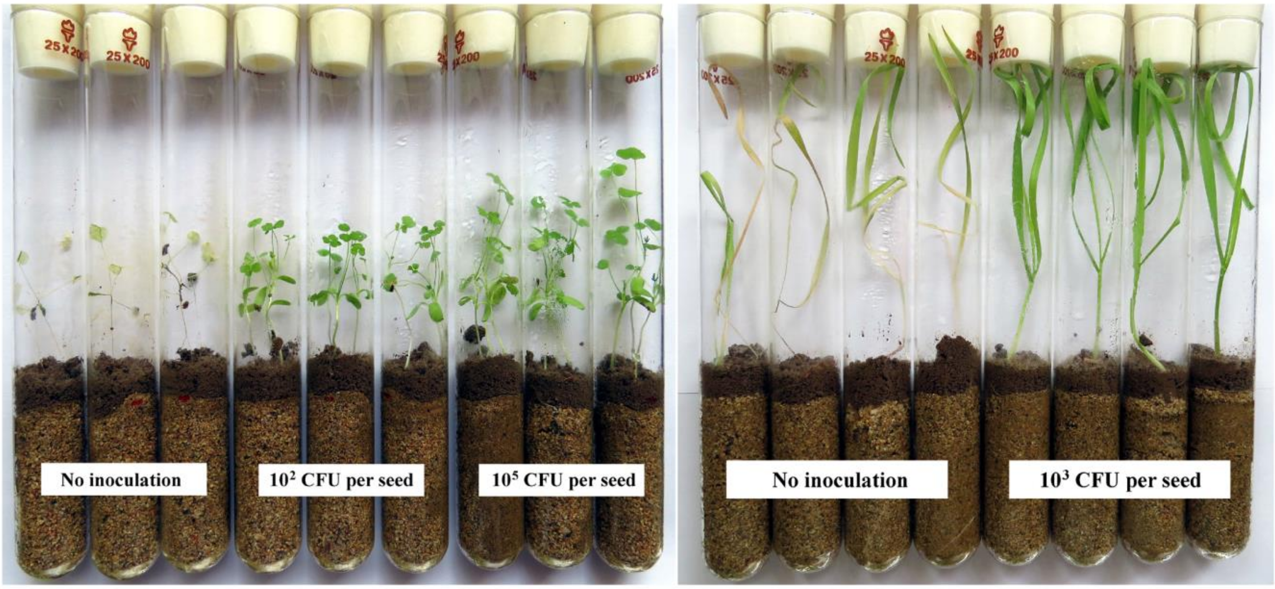

Fig. S1 A soil-sand assay for assessment of root colonization and atrazine degradation. CFU densities are the inoculum levels of *A. ureafaciens* DnL1-1.

Table S1 Quantification of atrazine (ATZ) and atrazine-2-hydroxy (HA) in the culture liquid of *A. ureafaciens* DnL1-1.

Parameter	Incubation, days							
	0	1	3	7	10	14	18	25
lg CFU/mL	5.59 ± 0.02	7.35 ± 0.03	7.28 ± 0.03	7.22 ± 0.03	7.11 ± 0.03	7.02 ± 0.02	6.88 ± 0.02	6.60 ± 0.03
ATZ concentration, ng/mL	2075.1 ± 175.0	≤0.096	<0.0192	<0.0192	<0.0192	<0.0192	<0.0192	<0.0192
ATZ molar concentration, nmol/L	9621.2 ± 811.4	≤0.445	<0.089	<0.089	<0.089	<0.089	<0.089	<0.089
lg ATZ molar concentration, nmol/L	3.98 ± 0.04	≤−0.35	<−1.05	<−1.05	<−1.05	<−1.05	<−1.05	<−1.05
HA concentration, ng/mL	0.480 ± 0.024	264.6 ± 11.3	199.9 ± 2.5	135.7 ± 1.8	93.0 ± 1.4	49.3 ± 0.2	23.7 ± 0.3	5.68 ± 1.22
HA molar concentration, nmol/L	2.43 ± 0.12	1341.5 ± 57.3	1013.5 ± 12.7	688.0 ± 9.1	471.5 ± 7.1	250.0 ± 1.0	120.2 ± 1.5	28.8 ± 6.2
lg HA molar concentration, nmol/L	0.39 ± 0.02	3.13 ± 0.02	3.01 ± 0.01	2.84 ± 0.01	2.67 ± 0.01	2.40 ± 0.01	2.08 ± 0.01	1.50 ± 0.04
HA percentage to the initial molar concentration of ATZ	0.025	13.94	10.53	7.15	4.90	2.60	1.25	0.30

Table S2 Quantification of atrazine-desethyl (DEA), atrazine-desisopropyl (DIA) and atrazine-desethyl-desisopropyl (DAA) in the culture liquid of *A. ureafaciens* DnL1-1.

Parameter	Incubation, days							
	0	1	2	3	4	7	11	21
lg CFU/mL	5.65 ± 0.02	6.97 ± 0.02	7.27 ± 0.03	NA	7.38 ± 0.03	7.18 ± 0.04	6.83 ± 0.02	6.82 ± 0.02
DEA concentration, ng/mL	956.3 ± 23.0	481.1 ± 19.9	4.41 ± 0.26	<0.0384	<0.0384	<0.0384	<0.0384	<0.0384
DEA molar concentration, nmol/L	5096.5 ± 122.6	2563.9 ± 106.3	23.5 ± 1.4	<0.204	<0.204	<0.204	<0.204	<0.204
lg DEA nmol/L	3.70 ± 0.02	3.41 ± 0.02	1.37 ± 0.03	<−0.69	<−0.69	<−0.69	<−0.69	<−0.69
DIA concentration, ng/mL	960.8 ± 42.5	897.73 ± 38.03	341.9 ± 6.2	71.8 ± 3.4	9.97 ± 1.57	5.98 ± 0.38	2.14 ± 0.06	2.26 ± 0.20
DIA molar concentration, nmol/L	5534.5 ± 244.7	5171.5 ± 218.8	1969.4 ± 36.0	413.7 ± 19.4	57.4 ± 9.1	34.4 ± 2.2	12.3 ± 0.4	13.0 ± 1.2
lg DIA nmol/L	3.74 ± 0.02	3.71 ± 0.02	3.29 ± 0.01	2.62 ± 0.02	1.76 ± 0.06	1.54 ± 0.02	1.09 ± 0.01	1.11 ± 0.04
DAA concentration, ng/mL	975.3 ± 28.1	1007.3 ± 37.3	997.2 ± 30.8	NA	NA	958.7 ± 34.6	NA	982.5 ± 9.4
DAA molar concentration, nmol/L	6700.7 ± 193.0	6920.5 ± 187.2	6851.5 ± 211.4	NA	NA	6586.5 ± 237.5	NA	6750.1 ± 64.1
lg DAA nmol/L	3.83 ± 0.01	3.84 ± 0.01	3.84 ± 0.01	NA	NA	3.82 ± 0.02	NA	3.83 ± 0.01

NA, not analysed.

Table S3 Degradation of atrazine by *A. ureafaciens* DnL1-1 in soil-sand tube assay.

Atrazine dose, µg/tube	Treatment	Plant	Atrazine recovered, µg/tube	Atrazine degradation
175.0	Atrazine-supplemented tubes	None	145.3±11.2	0 %
	Not planted not inoculated control	None	108.0±11.4	25.7 %
	Planted not inoculated control	Wheat	83.0± 1.6	42.9 %
	Inoculation with DnL1-1 (3.45 ± 0.04 lg CFU/seed)	Wheat	0.25±0.06	99.7 %
	Inoculation with DnL1-1 (6.67 ± 0.02 lg CFU/seed)	Wheat	0.37±0.14	99.8 %
3.5	Atrazine-supplemented tubes	None	3.14±0.55	0 %
	Not planted not inoculated control	None	1.91±0.37	39.2 %
	Planted not inoculated control	Wheat	2.15±0.53	31.5 %
	Inoculation with DnL1-1 (3.45 ± 0.04 lg CFU/seed)	Wheat	1.04±0.25	66.9 %
	Inoculation with DnL1-1 (6.67 ± 0.02 lg CFU/seed)	Wheat	0.28±0.21	91.8 %
17.5	Atrazine-supplemented tubes	None	12.44±1.61	0 %
	Not planted not inoculated control	None	7.05±1.00	43.3 %
	Planted not inoculated control	Alfalfa	7.88±2.26	36.7%
	Inoculation with DnL1-1 (2.08 ± 0.04 lg CFU/seed)	Alfalfa	3.42±1.38	72.5%
	Inoculation with DnL1-1 (4.32 ± 0.02 lg CFU/seed)	Alfalfa	3.04±0.85	75.6%

Table S4 Molar balance of atrazine and its degradation products in soil.

Analytes	Treatment					
	Blank soil	Blank soil + atrazine	Uninoculated unplanted control	Uninoculated planted (wheat) control	Inoculated unplanted control	*A.ureafaciens* DnL1-1 in association with wheat
Atrazine, nmol/g	0.0046±0.0006	27.4±2.4	14.8±1.8	15.8±1.3	11.1±2.2	0.580±0.039
HA, nmol/g	0.0050±0.0002	0.343±0.014	1.01±0.08	1.15±0.22	0.845±0.099	0.769±0.044
DEA, nmol/g	0.0025±0.0002	0.337±0.008	5.49±0.61	5.20±0.32	2.35±0.20	0.092±0.012
DIA, nmol/g	0.0015±0.0001	0.119±0.005	1.26±0.11	1.35±0.07	0.704±0.071	0.101±0.009
DAA, nmol/g	0.0029±0.0006	0.0062±0.0002	1.28±0.11	1.22±0.05	0.418±0.063	0.284±0.012
**Total analytes, nmol/g**	**0.0165±0.0017**	**28.205± 2.427**	**23.84±2.71**	**24.72±1.96**	**15.517±2.633**	**1.826±0.116**
Total analytes, %	0.058	100	84.52	87.64	55.01	6.47
Total degradates, nmol/g	0.0119±0.0011	0.805± 0.027	9.04± 0.91	8.92± 0.66	4.417± 0.433	1.246± 0.077
Total degradates, %	1.48	100	1122.7	1107.8	548,6	154.7
Total chlorinated degradates, nmol/g	0.0069±0.0009	0.462± 0.013	8.03± 0.83	7.77± 0.44	3.572± 0.334	0.477± 0.033
Total chlorinated degradates, %	1.49	100	1737.3	1681.1	772,8	103.2
Portion of chlorinated degradates in total degradates, %	58.0	57.4	88.8	87.1	80.9	38.3
Total chlorinated analytes, nmol/g	0.0115±0.0015	27.865± 2.413	22.83±2.63	23.57±1.74	14.672±2.534	1.057±0.072
Total chlorinated analytes, %	0.041%	100	81.9	84.6	52.7	3.79
Portion of chlorinated analytes in the total pollution, %	69.7%	98.8	95.7	95.3	94.6	57.9
